# Pain through the perspective of art and creativity: insights from the Unmasking Pain project

**DOI:** 10.3389/fpain.2023.1179116

**Published:** 2023-05-30

**Authors:** Mark I. Johnson, Paul Chazot, Frances Cole, Rosemary Cruickshank, Dawn Fuller, Charlotte Keyse, Balbir Singh, Adam J. Strickson, Ghazala Tabasam, Emma Tregidden, Kate Thompson, James Woodall

**Affiliations:** ^1^ Centre for Pain Research, Leeds Beckett University, Leeds, United Kingdom; ^2^ Pain Challenge Academy, Wolfson Research Institute for Health and Wellbeing, Durham University, Durham, United Kingdom; ^3^ Pain Academy, Live Well with Pain Ltd, Durham, United Kingdom; ^4^ INPUT Pain Management, Guy's and St Thomas’ NHS Foundation Trust, London, United Kingdom; ^5^ Space2 Leeds, Leeds, United Kingdom; ^6^ Balbir Singh Dance Company, Leeds, United Kingdom; ^7^ School of Performance and Cultural Industries, Faculty of Arts, Humanities and Cultures, University of Leeds, Leeds, United Kingdom; ^8^ Centre for Health Promotion Research, Leeds Beckett University, Leeds, United Kingdom

**Keywords:** pain, art, creativity, pain concepts, pain management

## Abstract

People struggle to tell their story of living with pain and when they do it is articulated in a way that may not be understood, heard or taken seriously. Unmasking Pain is an artist-led project that explored creative approaches to tell stories of life with pain. The project was led by a dance theatre company that specialises in storytelling and emotional experiences for players and audiences. The project involved artists and people living with ongoing pain co-creating activities and environments to curiously explore “oneself”, through imagination and creative expression. This article discusses insights and perspectives emerging from the project. The project revealed the power of art to make-sense of oneself with or without pain, and how art facilitates expression of complex inner experience and personal stories. People described Unmasking Pain as “explorative joy despite pain”, and “a new set of rules” that contrasts with those experienced during clinical encounters. We discuss how art has the potential to improve clinical encounters and promote health and well-being, and whether artist-led activities are an intervention, therapy, or something else. Pain rehabilitation specialists from the project described Unmasking Pain as “freeing-up thinking”, allowing conceptual thought beyond the biopsychosocial model of pain. We conclude that art has the potential to shift people living with pain from “I can't do, I am not willing to do it” to “Perhaps I can, I'll give it a go, I enjoyed”.

## Introduction

Tension between subjective pain and objective medicalised health care is long-standing. In clinical practice, a person's narrative about their complex pain experience is amalgamated or deconstructed into scalable items used in assessment tools. Consequently, people struggle to tell the story of their pain and when they do it is articulated in a way that is not consistently understood or is not heard or taken seriously.

Unmasking Pain was a pilot project to explore creative approaches for telling stories of life with persistent pain. Unmasking Pain sought to find a voice for people experiencing on-going pain through artistic collaboration with artists and artforms. The purpose of Unmasking Pain was to use a co-creative framework to explore various approaches to express stories, experiences, and challenges of living with pain. Our viewpoint was that exploring pain through the lens of art and creativity provides a more encompassing understanding of pain experience, overcoming the challenges of conceptualising and expressing pain.

This perspectives article discusses the views of people who were involved in the Unmasking Pain project, i.e., artists, pain specialists, support personnel, and people living with pain (participants). These people provided consent to use materials that documented their opinions and experiences during the project, including notes and recordings taken during briefing sessions, workshops, specific feedback focus groups, and informal interviews (in-person and on-line). The lead author (MJ) created a draft manuscript to summarise common themes and interesting perspectives from these sources. All authors revised draft manuscripts through an iterative process to ensure an accurate reflection of viewpoints.

## Synopsis of the project

A synopsis of the concept, design and delivery of the project is provided as Supplementary Material. In brief, artists collaborated with members of the project team and people with ongoing pain accompanying various conditions (participants), to design and deliver workshops to foster confidence in participants as human beings within a milieu of art and creative conversation. Artists worked with participants to articulate their story verbally and non-verbally, and to explore their story to help them understand themselves. Ethical approval was gained to embed two research studies in the project; a study to evaluate health-related data, including pain, and a phenomenology study to explore experiences via in-depth interviews.

During the project, participants engaged in various art activities including drawing, drumming, music making, writing, dance, drawing with pastels, clay modelling, puppetry, and nature walks. Some artists shared their own experience of living with pain and learning how to tell their stories, inspiring participants to find their own voice and gain self-confidence to unlock their own creativity. Art activities included mask-making of outer and inner facing selves, making personalised musical recordings with musicians, and taking personalised handmade puppets for a walk, to encourage participants to see themselves outside of themselves, i.e., a form of “creative treat”.

## Strategies in design and development of the project

### Vision of the artistic director

The vision was to use art to bring the worlds of artists and people living with pain together, to co-create a framework and process that worked for both. The Artistic Director handled the direction of the project through sensitivity in the moment to oversee and guide artists, distinguishing Unmasking Pain from art therapy. Strategic partnership building, sharing of vision and creative process, and gaining a sense of trust between everyone has been critical for success.

### Strategies of the artists

Early work of the artists involved developing sensitivity and trust by “circling around pain”, often not mentioning pain unless people wished to do so themselves. This was endorsed by participants from the outset in the phrase “Don't see me for my condition. See me for me”. Most workshops include an element of performance or the artist creating alongside the participants. Often, sessions started with the phrase “Sit back and relax, the artists are going to perform. You don't have to do anything. Just watch”. Through this process artists were not seen as invasive or threatening and participants quickly gained a sense of trust and confidence to engage in collaborative creative conversations.

Initially artists designed activities using colour, sound, and movement to creatively explore the severity and impact of pain. Musical improvisation was used to express inner experience through, for example, louder and faster rhythm to signify more intense pain and emotion. Figurative language such as “my muscles feel knotted” was creatively explored as “knots being an art or decorative element”, such as a plait of hair or “knots being a means of scaling experiences”, such as a complex tight knot representing severe pain, being difficult to untangle. This catalysed imaginative and creative discussion.

Activities based on the children's playground game “Hopscotch” were used to explore various themes, including a different approach to measure and scale pain from 1 to 10, and to explore the programme Ten Footsteps to live well with pain (https://livewellwithpain.co.uk/ten-footsteps-programme/). Hopscotch involves movement through a court of ten boxes drawn on the ground and artists demonstrated creative ways of interacting with the hopscotch court before inviting participants to join them through considerate and compassionate encouragement. Eventually, participants gained confidence to interact imaginatively with the hopscotch court either on their own or with others, catalysing creative discussions about supporting people with pain. For example, lying down on the entire hopscotch court to express the need to rest during a flare-up when pain intensity may be fluctuating from mild to severe.

## Experiences of participants: initial observations

A preliminary analysis of source materials suggested that participants experienced:

• Improvements in health-related data including pain and sleep, and reductions in medication
• Changes in sense-making, from the struggle of living with pain to developing a broader understanding of self and their own unique experience of “being”. This helps to create ways to express and acknowledge their story through engagement with the body, the world around and others
• Changes in emotion, including diffusion of anger, frustration, shame, fear, worry and lack of joy. This helps to strengthen pleasure, enjoyment, and a sense of relaxation
• Changes in ability and confidence to communicate, by having greater awareness of a state-of-mind and being more at ease with difficult emotions
• Changes in a sense of self and capacity to engage in new relationships with people, places, activities, and creative arts. Some participants returned to knitting or music making or had started doing other activities such as dog walking
• Changes through multifacet mediums, by exposure to a range of artists, people living with pain, artforms, colour, visual experiences, sensations of touch, diverse sounds; and by moving from passive to active engagement with creative artform to express self-identity by, for example, beginning to regularly play a new musical instrument.

Overall, participants reported feeling empowered to creatively explore themselves and this encouraged participants to be more physically and socially active, including discovering possibilities beyond the project such as taking up art activities, creative crafting, knitting, playing musical instruments, walking in nature, swimming, and visiting art galleries, museums, and historical buildings. Unmasking Pain achieved impact across cultural groups, and we attribute this to the visibility, diversity and culturally sensitive nature of the creative organisation and artists.

## Discussion: perspectives, insights, and implications

Without exception artists, pain specialists, support personnel and participants reported that involvement in the project affected how they think about pain. Here, we discuss insights and perspective-shifts arising from the project.

### The power of art to make-sense of oneself with or without pain

Evidence from meta-ethnography suggests that people living with long-term pain struggle to construct meaningful explanations for their suffering (1). For humans, “being alive” involves making sense of the relationship between sensations, emotions and thoughts arising within the body, and objects and events happening in the “external world”.

Chaplin contends that art enables contact with the external environment by acting as “… *a pre-reflective, nondiscursive mode of knowing, symbolizing, and being-in-the world.*” (2)p.1. Moreover, the symbolic practice of art enables humans to express emotional experiences that motivate re-interpretation and understanding of being in the world (2). For example, engaging in painting as an artform differs from painting a fence because that latter lacks significant symbolic action and interpretive meaning. Chaplin concludes that “… *the unique role of art [is] to be able to articulate or symbolize the world to the extent that it is affectively experienced. Put differently, art responds to the shapes, forms, and rhythms in the world to the extent that they can carry expressive meaning that resonates with the way we affectively experience the world*” (2) p. 10.

From an evolutionary perspective, art is a defining characteristic of the human species. The embodied nature of the practice and experience of art enables humans to project subjective sensory, affective, and cognitive experiences, such as pain, onto objects and events in the external world. Projecting subjective experience onto external objects facilitates a common understanding and sharing of bodily and spiritual states. Art can also bond values of justice, duty, social order, conflict, peace, and identity within and across diverse cultural backgrounds. A concept analysis of 85 studies by Kim and Lor revealed art activity “*… intrinsically motivated participants to create meaning for themselves and/or their health experiences. Such intrinsic motivation allows participants to experience growth, as well as the transformation of their health experiences*” (3) p. 8.

### The power of art to express the complexity of pain experience

Pain is a complex, dynamic, and multidimensional experience. Clinical practice relies on pain assessment that fuses elements of pain complexity into simplified generalisations or deconstructs pain complexity into fragmented items. Often these items are scaled and measured perpetuating an illusion of objectivity and evaluated solely within a clinical context. Pain experience is subjective. Self-report is a proxy of inner experience. Pain questionnaires splinter a person's experience and may decontextualize care (4, 5). The organic nature of art practice enables people to express holistic and contextualised experience with or without pain, offering novel ways of gaining insight to a person's state-of-being, self-identity and pain experience (6). Art empowers people during clinical consultation, aligning with the idea of “lay perspectives in healthcare” (7).

### The power of art to express personal stories

Unmasking Pain was conceived to enable people to share their self-identity, to tell the story of their pain, and for the story to be heard empathetically and taken seriously. Human social groups bond through story-telling and depriving humans of telling their stories is detrimental to health (8). Art enables people to express personal experiences and stories of bodily senses, emotions, thoughts, and journeys with or without words and syntax. Definitions of art vary over history and between cultures, although “*… the expression or application of human creative skill and imagination …*” (Oxford dictionary) is a fundamental characteristic of art. The educationalist, Sir Ken Robinson defined imagination as the ability to “bring to mind” things from the past, present, and future that are not immediately present to our senses; and creativity as the practical process of “applied imagination”, putting imagination to work to create output for others to see (9).

### The power of art to improve clinical encounters

People report having limited opportunities to tell their stories of living with pain and have them heard, especially in clinical environments (1). Bringing different artforms and media into conversations, opens new lexicons and modes of expression through which personal stories can be told, catalysing co-creation of meaning-making with family, friends, carers, strangers, and health care professionals. Evidence is growing that creatively expressing pain through visual artforms is likely to confer benefits to patients and improve clinical consultation (10, 11).

Healthcare professionals report having inadequate knowledge and skills to interpret verbal pain narratives, contributing to scepticism about a patient's self-report (12, 13). Hovey et al. (14) advocate expression of pain narratives through multiple artforms, including poetry and stories, to enable creative dialogues between patient and practitioner that foster empathy and understanding. Padfield et al. (10) argues co-creation of visual images can make the experience of pain visible, and that such images function as “transactional objects” that catalyse meaning-making and promote emotional disclosure by the patient, and non-verbal affiliative behaviour by the practitioner. Stilwell et al. (15) argues that art reveals to clinicians “how words might be received”, and this facilitates a deeper level of understanding of the fluid and interpretive nature of pain-related metaphors used by patients. Unmasking Pain demonstrates the power of art to create shared spaces to negotiate and co-create meaning between people experiencing pain and others, including artists and pain rehabilitation specialists. Art has the potential to facilitate education of health care professionals about the human condition.

### The power of art to promote health and well-being

In 2019, the World Health Organisation (WHO) published a scoping review that mapped evidence in the field of arts and health comprising over 700 individual studies and 200 literature reviews (16). The findings suggested that involvement in various types of artform can improve health and well-being in a variety of settings and for a variety of conditions including short and long-term pain (16).

The ethos of Unmasking Pain aligns with salutogenic models of health and well-being, i.e., that health is an outcome of everyday interactions between individuals and socio-ecological stressors (17). Salutogenic approaches focus on factors supporting well-being by maximising human potential, not just treating disease to return a person to “normality” (18). Increasingly, clinical care pathways and guidelines in the health sector are incorporating salutogenic concepts by recommendation of healthy lifestyle adjustment [e.g., National Institute of Health Care Excellence (NICE) guidelines for the management of chronic pain (19) and non-specific low back pain (20)]. The 2023 Global Awareness Campaign for the International Association for the Study of Pain (IASP) is “Integrative Pain Care” that emphasises non-drug, self-management care, and a person-centred focus (https://www.iasp-pain.org/advocacy/global-year/integrative-pain-care/). We advocate a role for art within health care frameworks, which raises debate how artist-focussed activities such as Unmasking Pain should be positioned within society, and health care.

### Art as an intervention, therapy, or something else?

Art-therapy is a type of psychological therapy delivered by trained art therapists/art psychotherapists. The uniqueness of Unmasking Pain is that it is led by artists who do not have therapeutic training and focus instead on engaging people with art in non-clinical contexts. This fosters interactions with the person not the pain, allowing the person to lead, take control, and have creative ownership, something that a clinician may find difficult to do. People living with pain often described the artist-participant encounter as “a different set of rules”. To paraphrase one participant during a workshop discussion “The pain clinic was good, but very clinical, and doesn't give you the social side. Unmasking Pain didn't feel rigid or like the artists were teaching you things. Most of the time, the artists had ideas of what they were going to do but the group of participants would take it somewhere else. And this didn't matter, and the artists were able to go with the new direction”. Such an approach is often impossible in clinical environments.

In 2021, Toye et al. (21) published a meta-ethnography of 195 qualitative studies that suggested health interventions for persistent pain should focus on validating pain through meaningful and acceptable explanations, validating patients by listening to and valuing their stories, and facilitating safe reconnection of patients with the social world by encouraging them to connect with a meaningful sense of self, be kind to themselves and to explore new possibilities for the future.

Unmasking Pain appears to satisfy these criteria. Kim and Lor (3), revealed four defining attributes of art-making as a health intervention; creation of art, creativity, self-expression, and distraction and helping people to adjust to living with pain by returning to meaningful activities (e.g., work hobbies, socialising etc.). Pain is known to reduce the sense of mastery and pleasure. Accessing artists can counter this by fostering positive emotionality, e.g., enjoyment, pleasure, relaxation, fun, control, mastery, empowerment, and achievement.

Artist-led interventions could be delivered by community-based creative and cultural organisations and venues and made available through social prescribing. Funders of such services will seek evidence of benefit and safety. The health sector values evidence of efficacy and harm via systematic reviews of randomised controlled clinical trials. Evidence to support the value of art interventions for pain is growing with systematic reviews providing tentative evidence that art and music therapy is beneficial, and safe, for people living with pain (22–27). Unmasking Pain is grounded in a “social aspect”, so using a conventional RCT paradigm to evaluate effectiveness is likely to be inadequate. In fact, the veracity of existing RCT evidence for pain treatments has been challenged (28). In 2021, NICE reported no relevant clinical studies comparing social interventions with standard care for the management of chronic primary pain (19).

### The power of art to challenge conceptual thinking about pain

Involvement in Unmasking Pain has instigated discussions among our pain specialists about tensions between subjective pain and the objective world of pain medicine dominated by a Cartesian neuro-mechanistic explanation of pain. Neilson argues that overly simplistic mechanistic models of pain encourage treatments focussed on “blocking and cutting” neural substrates (29). Conflation of nociception and pain has contributed to misnomers and fallacies in reasoning such as reification of pain (i.e., the myth that pain is a concrete (objective) “thing”) (30) resulting in conceptual misunderstanding (31). Pain scholars argue for conceptual shifts in models of pain that incorporate principles of meaning in relation to lived experience (32, 33), as well as contemporary understanding of neural processing that may influence pain qualia, such as nonlinearity, predictive processing and emergence (34). Debates about the biopsychosocial model of pain being conceptually narrow, fragmented, and dominated by biomedical paradigms has resulted in calls for broader perspectives (32, 35–38). Viewing pain through the lens of art and creativity can offer a more encompassing understanding of pain. For example, Agarwal used a medical humanism, social constructionist approach to develop an “ecology of wholeness” model for the person living with pain, in which art plays a key role in knowing, symbolising and healing the body and the self (39).

## Conclusion

People struggle to conceptualise and tell stories of their pain experience, and our perspective is that Unmasking Pain overcame this challenge by helping people living with pain move from “I can't do, I am not willing to do it” to “Perhaps I can, I'll give it a go, I enjoyed, … I am not alone”. This demonstrated the power of art to enable curious exploration of oneself, through imagination, creative expression, and explorative joy, enabling people to tell their stories and have them heard. To paraphrase Novalis (aka Friedrich von Hardenberg, 1772–1801) “*Art makes the familiar strange, and the strange familiar*”.

## Data Availability

The original contributions presented in the study are included in the article/**Supplementary Material**, further inquiries can be directed to the corresponding author.
