# Supplementary material for: Pain through the perspective of art and creativity: insights from the Unmasking Pain project
Source: Front Pain Res (Lausanne). 2023 May 30;4:1179116. doi: 10.3389/fpain.2023.1179116 (PMC10267741; doi:10.3389/fpain.2023.1179116)
Supplement: Supplementary file 1 [file Table1.docx]

Supplementary Material

Summary of the project

Date: 04 May 2023

This document provides further detail to supplement the section ‘Synopsis of the project’ provided in our article.

# Concept

Unmasking Pain was conceived and developed by an Artistic Director (Mr Balbir Singh) in conversation with pain rehabilitation practitioners involved in the development of the Ten Footsteps to live well with pain interactive online resource (i.e., Dr Frances Cole and Dr Paul Chazot <https://livewellwithpain.co.uk/ten-footsteps-programme/>).

Unmasking Pain started with the premise that people struggle to tell the story of their pain and when they do it is articulated in a way that is not consistently understood or is not heard or taken seriously. The idea was that people living with pain would work alongside artists to explore creative approaches and varied vocabularies (artforms) and forms of expression to tell stories of life with persistent pain. The ethos was to understand and celebrate the human condition through creative exploration of who we are in ourselves and how we live in the world around us. It was hoped that people with on-going pain would better understand themselves and what their story might be in new ways. Unmasking Pain was distinct because it was grounded in art rather than therapy, and artists engaged with people as people, not as ‘chronic pain sufferers’, an approach many clinicians and therapists can find difficult to do.

# Strategy

The Artistic Director’s (BS) vision was to build strategic partnerships and relationships of trust between artists, pain rehabilitation specialists, scientists and people living with pain to foster a sense of shared ownership of the project. Partners were:

- Balbir Singh Dance Company, an intercultural company making dance theatre
- Dancers living with on-going pain
- People living with on-going pain
- Space2, an arts and social change charity
- Pain rehabilitation specialists and scientists, from (i) the Wolfson Research Institute for Health and Wellbeing Pain Academy at Durham University, (ii) the Centre for Pain Research at Leeds Beckett University, (iii) Guy’s and St Thomas’ Hospital, and (iv) Live Well with Pain, an independent not-for-profit organisation to support people and practitioners manage long-term pain.

The framework for each workshop was created by a dance theatre company (Balbir Singh Dance Company) that specialises in literal or abstract storytelling and emotional experiences, for players and audiences. The source material for the artists were the participants living with pain, which included some artists with pain. Unmasking Pain would be an iterative process of sense-making by ‘tuning into our senses and the world around us’ to enhance ‘sweet spots for the senses’ (e.g., optimising pleasant sensations). It was hoped that creative dialogue and conversation with artists would allow people to maximise their purpose through growth, confidence building, and enjoyment in the moment.

# Delivery

Artists created workshops for people from different sociocultural backgrounds who were living with pain in towns or cities in the North of England. A programme of six, 90-minute workshops were delivered to a cohort of people living with pain associated with a variety of conditions including fibromyalgia, Parkinson’s Disease, arthritis, and cancer, and to a group of refugees with pain and the trauma of displacement and persecution. In addition, some stand-alone ‘pop-up’ workshops were delivered to groups of people with persistent pain. People were eligible to take part if they were willing to share their story, listen to the stories of others and prepared to take part in the creative process, i.e. ‘to try new things’, and to be open to scientific analyses.

The goal of each workshop was to foster confidence in people as human beings by enabling people to express, articulate, grow, have new encounters, new experiences and come together with other people in a milieu of art and creative conversation, i.e., a form of ‘creative treats’. Each workshop was designed to help people explore new vocabularies through exposure and immersion in different creative arts. This was to find different ways of understanding, processing, and making sense of themselves with and without pain. The locus of value creation was with participants who constructed outcomes that were relevant to themselves through interactions with artists.

Some artists were living with pain and were able to share their pain experience and personal stories with participants through non-verbal movements. Artists listened to a person’s story and expressed it in a creative way using their art form, generating repertoire through responsive, intuitive, and adaptive improvisation. In doing so, additional tools were provided for the person to articulate their story verbally and non-verbally, and to explore their story to help them understand themselves. Artists also introduced the possibility of representing different aspects of pain experience using different art forms, allowing participants to progress the expression of their pain. Artists and participants brought a range of experiences and perspectives to the workshops. This enabled creative practice that acknowledged a range of social issues that could influence perceptions, management and treatment of pain including race, age, cultural attitudes, and socioeconomic status.

# Documentation, Evaluation and Dissemination

Processes and products were documented by photographers and filmmakers and an end of project exhibition was co-created with participants. The exhibition comprised a multi-disciplinary installation that gave a voice to people living with pain and sought to influence pain narrative through holistic expression of experiences of living with pain. The installation was displayed in a shop in a local town and in health care settings including GP practices. The exhibition also included a tour of lecture-performances against a backdrop of the installation.

Evaluation of the project is iterative and involves gathering opinions and experiences from participants and project team members, and an analysis of data related to pain, mood, medication, and wellbeing. Two research studies, both ongoing, were embedded in the project: (i) A scientific study to investigate changes in behaviour (activity and sleep), medication, health, confidence in self-managing pain, and emotional arousal status using face imaging (morphology and thermal technology (1,2); and (ii) A phenomenological study to gain description and context about Unmasking Pain, from the perspectives of people who had experienced it.

# Workshop Activities

People participated in various art activities including drawing, drumming, music making, writing, dance, drawing with pastels, clay modelling, puppetry, and nature walks. People watched and listened to artists expressing pain through artforms such as dance expression movements and various types of music: ‘*We as artists cannot feel or express your pain, but we can help you show it, through for example, movement, where that pain could do this* [movement] *or go like that and do that* [movement rhythmically] *and then it slows down to this* [slow rhythmic movement]’.

Some artists shared their own experiences of living with pain and creative journeys of learning how to tell their stories, inspiring participants to find their own voice and gain self-confidence to unlock their own creativity. A notable example of this co-creative approach was ‘musical prescription’ where participants and artists produced a music treatment. Working with a tabla player and other musicians, participants chose sounds and music that inspired or relaxed them, that were then turned into their own personalised recordings. Various creative activities were devised to facilitate participants being able to see themselves outside of themselves. For example, ‘Taking your shoes for a walk’ involved naming your shoes (e.g. flip and flop, Janet and John, etc.) and having a conversation about yourself with your shoes whilst on a garden walk. Another example included a puppeteer designing and gifting personalised handmade puppets to each participant who then held conversations with themselves through their puppets. One participant dismantled their puppet so that they could carry it round in a handbag as it made them smile whenever they saw the puppet. Some participants and artists made a mask of their outer facing and inner facing selves at the beginning and again at the end of the project to capture the positive changes that occurred following the series of workshops.

# Status of the project

Project delivery is complete, and analysis of project data is ongoing. Recruitment has closed for both research studies and data is being analysed. Findings from these studies will be published in the future.

# References

1. Jiang, R., Chazot, P., Pavese, N., Crookes, D., Bouridane, A., and Celebi, M.E. (2022). Private Facial Prediagnosis as an Edge Service for Parkinson's DBS Treatment Valuation. IEEE J Biomed Health Inform 26, 2703-2713.

2. Kosonogov, V., De Zorzi, L., Honore, J., Martinez-Velazquez, E.S., Nandrino, J.L., Martinez-Selva, J.M., and Sequeira, H. (2017). Facial thermal variations: A new marker of emotional arousal. PLoS One 12, e0183592.

# Project Acknowledgements

## Artists

Bobak Champion, Kali Chandrasegaram, Ford Collier, Mansi Dabral, Louise Grassby, Vilmore James, Natasha Joseph, Madhura Karandikar, Sarah Mason, Aniruddha Mukherjee, Sarah Partridge, Devika Rao, Balbir Singh, Chris Speyer, Adam Strickson, Sam White, Jenny Wilson

## Project Support

Holly Amos, David Andrassy, Mark Baker, Paul Floyd Blake, Susan Burns, Gareth Dakin,

Julian Germain, Anna Harpin, Tim Ingram, Malcolm Johnson, Jordan Mereil, Ezekiel Oliveira, Bisakha Sarker, Tim Smith, Elia Tome, Nathan Towers, Tammy Tsang, Jess Williams, Anamaria Wills, Karol Wyszynski
